# *Coxiella burnetii* dormancy in a fatal ten-year multisystem dysfunctional illness: case report

**DOI:** 10.1186/s12879-016-1497-z

**Published:** 2016-04-18

**Authors:** Olga A. Sukocheva, Jim Manavis, Tuck-Weng Kok, Mark Turra, Angelo Izzo, Peter Blumbergs, Barrie P. Marmion

**Affiliations:** Q Fever Research Group (1993–2009), Hanson Institute, Adelaide, South Australia; Centre for Neurological Diseases, SA Pathology, Adelaide, South Australia; School of Biological Sciences, University of Adelaide, Adelaide, South Australia; Microbiology and Infectious Diseases Laboratory, SA Pathology, Adelaide, South Australia; Colorado State University, Colorado, USA; School of Health Sciences, Flinders University, Bedford Park, South Australia

**Keywords:** Q Fever, Antigen persistence, Chronic fatigue syndrome

## Abstract

**Background:**

In a previous study of a Q fever outbreak in Birmingham, our group identified a non-infective complex of *Coxiella burnetii* (*C.b.*) antigens able to survive in the host and provoked aberrant humoral and cell-mediated immunity responses. The study led to recognition of a possible pathogenic link between *C.b.* infection and subsequent long-term post Q fever fatigue syndrome (QFS). This report presents an unusually severe case of *C.b.* antigen and DNA detection in post-mortem specimens from a patient with QFS.

**Case presentation:**

We report a 19-year old female patient who became ill with an acute unexplained febrile encephalitis-like illness, followed by increasingly severe multisystem dysfunction and death 10 years later. During life, extensive clinical and laboratory investigations from different disciplinary stand points failed to deliver a definitive identification of a cause. Given the history of susceptibility to infection from birth, acute fever and the diagnosis of “post viral syndrome”, tests for infective agents were done starting with *C.b.* and *Legionella pneumophila*. The patient had previously visited farms a number of times. Comprehensive neuropathological assessment at the time of autopsy had not revealed gross or microscopic abnormalities.

The aim was to extend detailed studies with the post-mortem samples and identify possible factors driving severe disturbance of homeostasis and organ dysfunction exhibited by the course of the patient’s ten-year illness. Immunohistochemistry for *C.b.* antigen and PCR for DNA were tested on paraffin embedded blocks of autopsy tissues from brain, spleen, liver, lymph nodes (LN), bone marrow (BM), heart and lung. Standard H&E staining of brain sections was unrevealing. Immuno-staining analysis for astrocyte cytoskeleton proteins using glial fibrillary acidic protein (GFAP) antibodies showed a reactive morphology. C*oxiella* antigens were demonstrated in GFAP immuno-positive grey and white matter astrocytes, spleen, liver, heart, BM and LN. PCR analysis (COM1/IS1111 genes) confirmed the presence of *C.b.* DNA in heart, lung, spleen, liver & LN, but not in brain or BM.

**Conclusion:**

The study revealed the persistence of *C. b.* cell components in various organs, including astrocytes of the brain, in a post-infection QFS. The possible mechanisms and molecular adaptations for this alternative *C.b.* life style are discussed.

## Background

Post infection fatigue syndrome has been described in patients exposed to facultative intracellular bacteria, including *Coxiella burnetii (C.b.)* - the causative agent of Q fever, as well as viruses [1]. The highly infectious (*ca.* one organism) rickettsia-like intracellular bacterium infects and multiplies in macrophages. In a previous report of a Q fever outbreak in Birmingham, our group identified a non-infective complex of *C.b.* antigens able to survive in the host and provoked aberrant humoral and cell-mediated immunity responses [2, 3]. The study led to recognition of a possible pathogenic link between infection and subsequent long-term post Q fever chronic fatigue syndrome (QFS).

We report a 19-year old female patient (coded initials BI) who became ill with an acute unexplained febrile encephalitis-like illness, followed by increasingly severe multisystem dysfunction and death ten years later (in 1996). During life, extensive clinical and laboratory investigations from different disciplinary stand points failed to deliver a definitive identification of a cause, but descriptive diagnoses, such as post infection fatigue syndrome, or just before death, Behçet’s syndrome, were proposed. During her last 10 years, BI presented with severe fluctuating headaches, frequent dizziness, fever 40C+, recurrent episodes of extensive pharyngeal ulcerations, muscular pain, persistent fatigue, joint pains, myoclonic seizures, quadriparesis, symptoms suggestive of meningism (neck rigidity and photophobia), bulbar paralysis and a range of gastrointestinal tract symptoms including abdominal pains, nausea, diarrhoea, bloating, oesophageal spasms as well as weight loss. It was noted that before the “encephalitis” there had been a history of inadequate, slow immune resolution on contracting various childhood infections.

At autopsy, standard histo-pathological methods revealed few abnormalities gross, or microscopic - an ulcer of the hard palate and very sparse patchy chronic inflammatory cell infiltration close to the atrio-ventricular node conducting system of the heart. Levels of inflammatory markers such as C-reactive protein (CRP) were within normal range, but with increased erythrocyte sedimentation rate. The negative organ and tissue profile was in sharp contrast to the severity of the symptomatic effects during life that included abnormal disabling fatigability, transient loss of consciousness (“blackouts”), loss of control over electrolyte balance and unexplained tissue oedema.

In view of BI’s early history of abnormal susceptibility to infections, her acute fever and ‘encephalitic’ symptoms with a diagnostic label of severe “post viral infection fatigue syndrome”, we suggested that her chronic persistent and severe multisystem disability might be an incidental systemic side effect (“bystander damage”) of the specialised effector mechanisms, immune mediators and other gene products of facultative intracellular bacteria.

A major challenge remained that despite intense and dedicated investigative efforts from various medical sub-disciplines it had not been possible to identify definitively the factors driving the severe disturbance of homeostasis and organ dysfunction exhibited by the course of BI’s 10-year illness. In anticipation that a changed paradigm for post infection and related fatigue states would eventually emerge, BI’s family had retained the paraffin wax-embedded or fixed slices of blocks of autopsy tissues from the patient’s brain, spleen, liver, lymph nodes (LN), bone marrow (BM), heart, lung and other organs. The post mortem samples were submitted by the family for examination by extended techniques to search for possible changes in the brain using immune-cytochemical markers for astrocytes and microglia. In the course of examining the paraffin wax-embedded tissues for possible neuropathology, prior Q fever infection was considered. BI had previously visited farms a number of times during childhood (see review [4]). Q fever antigens or specific antibodies had not been previously tested. This led to extended tests with staining for Q fever antigens in the paraffin wax-embedded tissues. This report presents the immunohistochemical and PCR findings of *C.b.* antigens and DNA respectively in several organs, including astrocytes, from the patient’s post mortem samples and discusses the association between persistence of the antigens, albeit non-infective, and QFS.

## Case presentation

### *C.b.* antigen detection (Immunocytochemistry)

Post-mortem samples were collected, formalin-fixed (4 %, v/v), and paraffin-embedded. Sections (5 μm) of paraffin-embedded tissues were cut and mounted on APES coated (Superfrost plus) tissue slides and de-paraffinised according to standard protocol. Control healthy (disease free) brain samples were collected from car accident trauma patient and used as an adequate standard control [5]. Slides were stained with routine Haematoxylin and Eosin (H&E) for initial microscopic examination. For *C.b.* antigen detection, sections were pre-treated in citrate buffer (10 mM; pH 6.0) and heated by microwave and then allowed to cool to room temperature [5]. Antisera and negative sera were diluted in PBS. Immuno-staining was performed on selected slides with primary antibodies for the following markers: (1) *C.b.* Phase I and II antigens (rabbit polyclonal) or mouse Phase I monoclonal (a gift from CSL, Australia) [2, 3]; (2) polyclonal anti-GFAP (Santa Cruz Biotechnology); (3) monoclonal anti-lba-1 (Abcam). Negative controls consisted of sections immunostained as described below, but instead of overnight incubation with primary antibodies these were incubated with tris-buffered saline only (*data not shown, but available*). After washes in PBS/0.05 % Tween-20 (Fisher Scientific), sections were incubated with 1:100 dilutions of anti-rabbit or anti-mouse secondary antibodies conjugated to fluorescein isothiocyanate (FITC) or phycoerythrin (PE) (Jackson lmmunoResearch Laboratories Inc). Nuclei were stained with Hoechst 33342 (Invitrogen). Fluorescence images were acquired and processed using the analysis software Olympus Soft Imaging System. For the images where comparisons of staining intensities were made, all images were acquired using the same attenuator and exposure settings.

### *C.b.* DNA test (PCR)

DNA target nucleic acid sequences in tissue samples were tested by two separate PCR assays, one targeting the 27 kDa outer membrane protein (COM1) and the other the insertion sequence (IS1111) [6]. These were tested with the DNA extracted from 2 paraffin-embedded tissue sections (10 mm thick) using Proteinase K 2 mg/ml, SOS 0.5 % w/v, and Qiagen (Hilden, Germany) DNA blood kit. Samples were amplified as described previously [6]. To minimize possible false positives, DNA extraction and PCR were performed carefully according to guidelines described previously [7, 8]. A PCR result was considered negative (NEG, Table 1) when the Ct value was ≥37. In a real time qPCR assay (quantitative PCR), the cycle threshold (Ct) value is calculated to be the number of amplification cycles required for accumulated fluorescence signals in the reaction to exceed the background value. The higher the Ct value, the lower is the amount of amplified target nucleic acid sequence (Sequence Detection Systems v2.3 software - Applied Biosystems).

**Table 1:** Quantitative PCR (qPCR) tests for *C.b.* DNA (IS1111a sequence) with patient BI’s paraffin-embedded tissues were tested in two PCR cyclers – RotorGene 1.7.73 (Corbett Research) and Roche LightCycler 4.05 (4.0.0.23)

Patient’s post mortem specimens	Ct - RotorGene	Ct - LightCycler
Heart	23.34	25.65
Lung	36.37	36.46
Brain	NEG	NEG
Bone marrow	NEG	NEG
Spleen/Liver	25.15	27.08
Lymph nodes	22.47	24.80
Control spleen	NEG	NEG
Control brain	NEG	NEG

The RotorGene Ct values shown are average of duplicate tests and LightCycler values from single tests. Non-template controls (NTC) were placed in between the samples and were negative (Ct >37). Thick sections of the post mortem tissues – for PCR tests - were cut with high level of precaution to minimize possible cross contamination at the Centre for Neurological Diseases (SA Pathology, South Australia)

### Investigation of patient BI’s brain samples with immunohistochemical staining for astrocyte and distribution of *C.b.* antigen

**Fig. 1:**
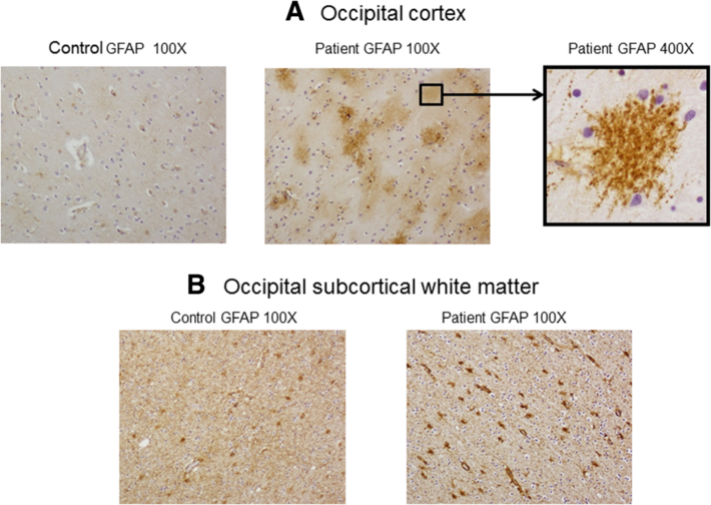
Assessment of Patient BI and control astrocyte GFAP immune-staining in occipital cortex and occipital subcortical white matter using peroxidase staining technique and anti-GFAP antibodies as described in Methods. **a** Peroxidase staining with GFAP in samples from BI occipital cortex showing a reactive astrocyte with numerous “beaded” processes (400X). **b** Peroxidase staining with GFAP in BI samples from occipital subcortical white matter showing increased GFAP immune-staining and a reactive phenotype. Representative photographs are shown

Given the history of susceptibility to infection from birth, the acute fever in 1986 and the diagnosis of “post (viral) infection syndrome”, tests for infective agents were done starting with *C.b.* and *Legionella pneumophila* (*L.p.*). Patient BI had previously visited farms a number of times. Previous comprehensive neuropathological assessment at the time of autopsy in 1986 had not revealed gross or microscopic (H & E staining) abnormalities. The H&E examination was repeated on fresh sections of brain from the patient and an unrelated control brain but no abnormalities were detected (data not shown). Immuno-peroxidase staining for glial fibrillary acidic protein (GFAP) showed prominent immune-staining of astrocytes and “beading” of the fibrillary processes compared with control brain sections which did not show such striking changes (Fig. 1a and b). Increased GFAP immuno-staining may be seen in many conditions that may be a non-specific reactive response of the astrocytes [9, 10].

**Fig. 2:**
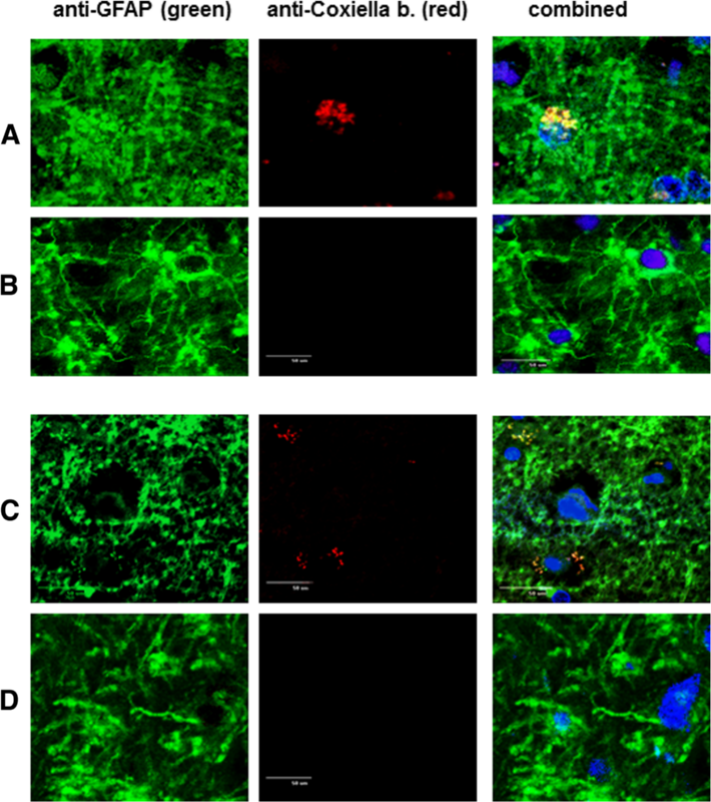
Patient BI and control brain samples from occipital cortex grey matter and occipital subcortical white matter were analysed using confocal microscopy for presence of *C.b.* antigens and co-stained for GFAP. Tissues were stained with anti-GFAP primary antibodies/FITC (*green*)-conjugated secondary antibodies; and co-stained with anti-*C.b.* primary antibodies/PE-conjugated (*red*) secondary antibodies as described in Material and Methods. Hoechst (*blue*) was used to stain cell nuclei. Magnification was set at 400X. **a** BI’s occipital subcortical white matter; **b** control patient’s occipital subcortical white matter; **c** BI’s occipital cortex grey matter; **d** control patient’s occipital cortex grey matter

Astrocytes in the occipital cortex and subcortical white matter from patient BI and control brain tissue were stained using GFAP antiserum with green-fluorescent (FITC) secondary antibodies, co-stained with Texas-red labelled monoclonal *C.b.* Phase 1 antiserum [2, 3] and viewed with confocal microscopy (Fig. 2). Astrocytes in the white and grey matter from patient BI (Fig. 2a and c, white and grey matter respectively) and control brain tissue (Fig. 2b and d) stained with anti-GFAP (green). Specific *C.b.* stained red and showed co-localization (yellow) with GFAP (green) protein (Fig. 2a). Staining with *C.b.* antisera revealed *C.b.* antigen complex in patient’s astrocytes, supporting the possible involvement of Q fever infection in the illness. Note that only a minority of astrocytes contained the *C.b.* Phase 1 antigen complex. *C.b.* antigens were also detected in cells from hippocampal white matter using dual staining with anti-GFAP and confocal microscopy (data not shown). White and grey matter staining for *C.b.* was negative in a control (uninfected) brain. Microglia, the innate immune cells mediating inflammatory responses in the CNS [10–12] were identified using the specific Iba-1 marker [13, 14]. Double immune-staining of the Iba-1 reactive cells with *C.b.* antisera was negative (data not shown).

### Distribution of *C.b.* antigens in other tissue samples from patient BI

**Fig. 3:**
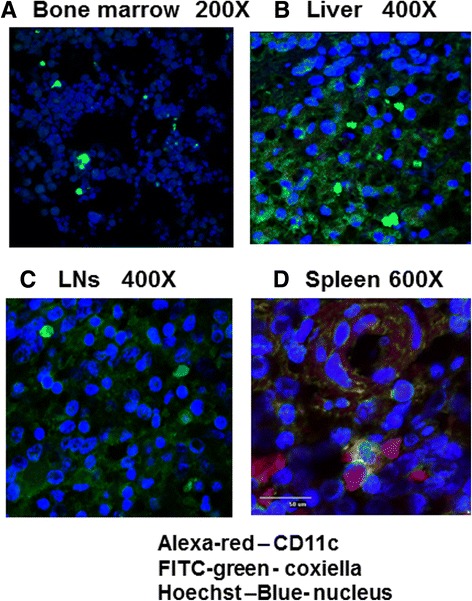
Presence of *C.b*. complexes in patient BI’s lymphoid tissues were assessed using confocal immunostaining assay: **a** Bone marrow, **b** Liver, **c** LNs, **d** Spleen. Alexa-red was used to identify CD11c (dendritic cells - only for D). *C.b.* specific antibody and FITC (*green*) secondary antibody were used to detect presence of C.b. and Hoechst (*blue*) stain defines nuclei

**Fig. 4:**
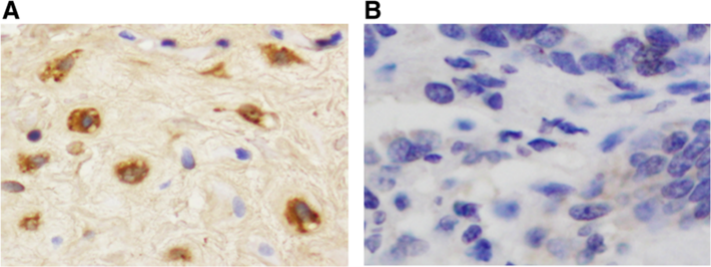
**a** Immuno-peroxidase staining of myocardium - close to mitral valve - with specific monoclonal antibodies for presence of C.b. antigens X400. **b** Negative control

Tissue samples from several lymphoid organs and those previously determined as targets for *C.b.* were further stained using specific antisera. Figures 3 and 4 show *C.b.* Phase 1 LPS antigens detected in the macrophages of the patient’s bone marrow (Fig. 3a), liver (Fig. 3b), lymph node (Fig. 3c), spleen (Fig. 3d) and heart (Fig. 4). Strong staining with the monoclonal antibody to *C.b.* Phase 1 LPS antigen was detected in both immuno-peroxidase (see Fig. 4) and IFA reactions (data not shown). However, the pattern was markedly different to that commonly described in Q fever endocarditis (early or late stage) where the valve lesions show fibrosis and calcification in the late stage [15]. The paucity of inflammatory cells including monocyte/macrophage lineage and the rapid decline of infective coxiella leaving antigen LPS complex has frequently been remarked, as are low levels of genomic DNA [16].

### *C.b.* DNA results

PCR tests for *C.b*. genomic DNA sequences (COM1 and IS1111a genes [6]) in separate tests did not detect the presence of specific DNA in patient BI’s brain, but nevertheless revealed substantial *C.b.* DNA in heart, lung, spleen and liver (Table 1) - in agreement with the results found during IFA and peroxidase staining with *C.b.* Phase 1 antisera (Figs. 3 and 4). PCR tests for COM1 and IS1111a genes showed similar results but only Ct values for the latter gene are shown in Table 1. The negative PCR result in the brain tissues may be due to DNA levels below the limit of detection (10–20 copies/ml, unpublished data).

## Conclusions

The scope and overall interpretation of our report on the re-evaluation of the fixed, paraffin-embedded tissue samples taken at patient BI’s autopsy are necessarily limited by the absence of diagnostic results of any serological or other tests for candidate infective organisms at the time of the “viral encephalitis” in 1986 (see review [17]). Nevertheless, taken together, the extended laboratory tests with *C.b.* specific monoclonal antibodies and PCR (COM1 and IS1111a genes) on a range of post mortem specimens suggest that the most compelling and coherent explanation of BI’s illness from 1986 to 1996, is one of a severe attack of primary Q fever and a subsequent multisystem organ dysfunction with dissemination of the coxiella throughout the body, ending in 1996 with cardiac and cerebral dysfunction i.e.*,* a complex, severe idiopathic illness labelled descriptively at the time as “post (viral) infection fatigue syndrome” (PIFS).

An epidemiological and clinical association between laboratory-confirmed primary acute Q fever infection and a post infection fatigue syndrome was described in 1996/8 in meat workers and farmers in Australia and in patients in the wind-borne sheep-associated Q fever outbreak in the non-occupationally exposed populations of South Birmingham UK [18, 19]. At first the post Q fever syndrome (QFS) tended to be interpreted as a psychogenic response by patients to a chronic debilitating illness. Since then, however, the association has been described in many countries. For example, an independent follow up of patients in a goat-associated Q fever outbreak in Alberta, Canada included a patient with severe unresolved cerebral symptoms but *C.b.* was not isolated from the CNS [20]. The recent major prevalence (>4000 cases) of goat-associated Q fever in the Netherlands, allowed well-controlled surveys for “post infection fatigue syndromes” and again confirmed prevalence of QFS and vascular complications after an initial infection [21].

Some of the scepticism and difficulty of acceptance of QFS may have rested on a mistaken assumption that the model of infection with the small intracellular bacterium, *Coxiella burnetii,* would follow a familiar set of characteristics of invasion, bacteraemia, inflammatory responses, finally resolution with rising antibody levels, acquired cellular immune responses and elimination of the invading organism [2]. Investigations of QFS by the Q fever Research Group in Adelaide (Australia) have not supported this simple model [2, 3]. Notably, long term persistence in the host of various components of the coxiella antigens shown e.g. LPS Phase I antigen, coxiella genomic DNA sequences and low level lgG class antibody in the human host to *C.b.* Phase I & II antigens were often present in QFS patients more than 10 years after the original infection [3]. On the other hand, in perplexing contrast, efforts to demonstrate consistent presence of the readily serial cultivable coccobacilliary form of the coxiella (small cell variant, SCV) in such materials have mostly failed [2, 3, 18–20].

The SCV, the infectious particle, is found in the environment and is metabolically inactive. When internalized into a cell the SCV is transformed into the metabolically active large cell variant (LCV) [22]. The LCV develops in response to acidification of the endosome and is associated with the development of the parasitophorous vacuole (PV) by the host cell that allows the organism to replicate. There is evidence to suggest that the highly acidic PV is regulated by the LCV and provides an excellent environment for it to grow. Further studies will be required to determine the relationship of the SCV with the PV [23, 24].

In the acute phase of Q fever, *C.b.* SCV is widely distributed in the host but in general they do not persist as they transition to the LCV form. In contrast non-infective *C.b.* components - complexes of Phase 1 LPS, antigens, DNA sequences or insertion elements have been detected in PBMC, bone marrow aspirates or valve vegetations [2, 3, 18, 19]. Such residual complexes of coxiella cell components persisted for 12–17 years after infection in the patients in the Birmingham UK Q fever outbreak [3]. Despite some partial “immune” responses the *C.b.* materials are not cleared and IL-2 is down-regulated, IFN-γ responses distorted and IL-6 responses are enhanced [25, 26]. The effect is supported by robust ability of *C.b.* to interfere with pathogen-initiated apoptosis upon infection of mammalian cells [27, 28] and by the activation of type IV intracellular organism effector repertoire [29, 30].

In summary, the recorded history, clinical findings and indices for patient BI, and the series of reports on QFS patients from the Adelaide Q fever Research Group in Australia and Birmingham UK, support a hypothesis that there is an association to be tested between persistence of *C.b.* components - as described above - the modified macrophage as a PV. The terms PIFS or QFS - while correctly labelling a major clinical presentation - do not do justice to the range of different effects or multiple host organ systems involved, particularly as illustrated in an unusual and exceptionally severe case such as patient BI.

### Ethics approval and consent

Not applicable.

### Consent for publication

Written informed consent was obtained from the patient’s parent for publication of this Case Report. A copy of the written consent is available for review by the Editor of this journal.

### Availability of data and materials

All data supporting the findings are within the manuscript.

## Abbreviations

BM: bone marrow
C.b.: Coxiella burnetii
FITC: fluorescein isothiocyanate
GFAP: glial fibrillary acidic protein
IFA: immunofluorescent antibody
IFN: interferon
LCV: large cell variant
LN: lymph nodes
LPS: lipopolysaccharide
PCR: polymerase chain reaction
PIFS: post (viral) infection fatigue syndrome
PV: parasitophorous vacuole
QFS: Q fever fatigue syndrome
SCV: small cell variant
